# A wide diversity of viruses detected in African mammals involved in the wild meat supply chain

**DOI:** 10.1371/journal.ppat.1013643

**Published:** 2025-12-29

**Authors:** Mare Geraerts, Sophie Gombeer, Casimir Nebesse, Douglas Akaibe, Dudu Akaibe, Pascal Baelo, Anne-Lise Chaber, Philippe Gaubert, Guy-Crispin Gembu, Léa Joffrin, Anne Laudisoit, Nicolas Laurent, Herwig Leirs, Claude Mande, Joachim Mariën, Steve Ngoy, Jana Těšíková, Ann Vanderheyden, Rianne van Vredendaal, Erik Verheyen, Sophie Gryseels

**Affiliations:** 1 Evolutionary Ecology group, Department Biology, University of Antwerp, Wilrijk, Belgium; 2 Virus Ecology Unit, Department of Biomedical Sciences, Institute of Tropical Medicine Antwerp, Antwerp, Belgium; 3 Biodiversity and Conservation Biology, Swiss Federal Institute for Forest, Snow and Landscape Research WSL, Birmensdorf, Switzerland; 4 OD Taxonomy and Phylogeny (BopCo), Royal Belgian Institute of Natural Sciences, Brussels, Belgium; 5 Centre de Surveillance de la Biodiversité (CSB), University of Kisangani, Kisangani, Democratic Republic of the Congo; 6 School of Animal and Veterinary Sciences, University of Adelaide, Adelaide, South Australia, Australia; 7 Centre de Recherche sur la Biodiversité et l’Environnement (CRBE), Université de Toulouse, CNRS, IRD, Toulouse INP, Université Toulouse 3 – Paul Sabatier (UT3), Toulouse, France; 8 CIIMAR/CIMAR, Interdisciplinary Centre of Marine and Environmental Research, University of Porto, Terminal de Cruzeiros Do Porto de Leixões, Matosinhos, Portugal; 9 Nature Health Global, Tallman, New York, United States of America; 10 OD Taxonomy and Phylogeny, Royal Belgian Institute of Natural Sciences, Brussels, Belgium; 11 Department of Environmental Sciences, Open Universiteit, Heerlen, The Netherlands; University of New Mexico School of Medicine, UNITED STATES OF AMERICA

## Abstract

The processes involved in acquiring, trading, preparing, and consuming wild meat pose significant risks for the emergence of zoonotic infectious diseases. Several major viral outbreaks have been directly linked to the wild meat supply chain, yet our knowledge of the virome of many mammals involved in this chain remains limited and disproportionately focused on certain mammalian taxa and pathogens. Here, we present the findings of a metagenomic viral screening of 101 mammalian specimens belonging to 28 wild African species and one domesticated species, all traded for their meat. The study focuses on tissue and swab samples collected in various regions in the Democratic Republic of the Congo and in Brussels, Belgium. A total of sixteen virus strains were detected, belonging to the families *Arteriviridae*, *Retroviridae* and *Sedoreoviridae* (primates), *Picobirnaviridae* (primates and rodents), *Picornaviridae* (rodents), *Hepadnaviridae* (hyrax), *Orthoherpesviridae* (artiodactylid and carnivore) and *Spinareoviridae* (carnivore). Several strains were detected in mammalian hosts for the first time, expanding their host range and genetic diversity. Of note is the presence of viruses genetically related to recognised zoonotic pathogens, i.e., human picobirnavirus (*Orthopicobirnavirus hominis*) (primates and rodents), simian foamy viruses (*Simiispumavirus*) (primates), and rotavirus A (*Rotavirus alphagastroenteritidis*) (primates). The presence of these viruses in primates is concerning as non-human primates are phylogenetically closely related to humans, which can facilitate interspecies viral transmission. These findings underscore the high diversity of mammalian viruses and the potential risk of human infection through cross-species transmission during close interactions with wildlife in the wild meat supply chain.

## Author summary

The wild meat supply chain represents a critical interface between humans and wildlife, posing significant risks for zoonotic disease emergence. Despite previous links between viral outbreaks and the wild meat supply chain, our understanding of the virome of many traded mammalian species remains incomplete. In this study, we conducted a metagenomic viral screening of 101 mammals from 28 wild African species and one domesticated species, all traded for their meat in the Democratic Republic of the Congo or Brussels, Belgium. We identified sixteen virus strains across multiple viral families, some of which were detected in these particular mammalian species for the first time. Notably, we found viruses closely related to known zoonotic pathogens, including human picobirnavirus, simian foamy viruses, and rotavirus A—with primates, a taxonomic group closely related to humans, serving as hosts. These findings highlight the vast viral diversity present in species involved in the wild meat trade and reinforce the potential for cross-species transmission during handling, trade, and consumption. Our study contributes to the growing understanding of wildlife viromes and provides crucial insights into the risks associated with the wild meat supply chain, emphasising the need for enhanced surveillance and mitigation strategies to prevent future zoonotic spillovers.

## Introduction

Most human infectious diseases originate from animals, including domesticated and wild animals [1,2]. Especially in tropical regions, wild mammals are an important reservoir of zoonotic pathogens [3]. One significant path for the transmission of these pathogens from wildlife to humans is through activities involved in the supply chain of wild meat, defined here as meat from non-domesticated mammals — also commonly referred to as “bushmeat” in tropical regions [4]. These activities include intimate human-wildlife interactions, such as hunting, butchering, selling, cooking, and consumption, which facilitate the transmission of zoonotic pathogens to humans [5–7]. Between 1940 and 2021, most recorded zoonotic spillover events associated with the wild meat supply chain occurred in Africa, where viruses were the most frequently reported pathogens [4]. Over a quarter of mammalian species involved in the wildlife trade, including the trade of wild meat, harbour 75% of known zoonotic viruses [1,2,8]. Notable examples of viral disease outbreaks that have been linked to contact with wild meat are Mpox [9], Ebola disease [10], SARS, and COVID-19 [11,12].

In many parts of the Afrotropics, hunted wildlife traditionally serves as a source of protein, micronutrients, and income for many rural communities [13,14]. Rapid population growth and urbanisation, the availability of firearms, and the increased rainforest accessibility through the expansion of road networks and logging, have led to unsustainable levels of commercial hunting of wildlife species [15,16]. Thus, in addition to public health concerns, the hunting of wildlife for their meat poses a significant threat to the conservation of tropical animals, driving many species towards extinction and potentially disrupting ecosystem services and functioning [15,17,18]. The urban demand is mainly driven by the taste of wild meat, cultural connotations, and the perception that wild meat is more ‘pure’ and healthier than domesticated animals, and many people are willing to pay higher prices than for domesticated meat [6,15,19]. As a result, wild meat consumption has well expanded beyond the geographical area where the meat is harvested and the high demand from international diaspora leads to a further intercontinental spread of these exotic animal products and the potential pathogens that they may carry [19–22]. In Europe, high quantities of unregulated wild meat are imported. For example, in Belgium, an estimated 3.9 tonnes of wild meat enters illegally each month via Brussels Airport, primarily originating from sub-Saharan Africa, with the Democratic Republic of the Congo (DRC) as the main source [20,23].

Our understanding of the wildlife virome of African mammals and their meat is limited and skewed towards species that are feasible to sample in the wild and those with a history of reported zoonotic disease emergence [24–27]. These include small mammal species with large populations, such as common gregarious bats, rodents, and shrews. Consequently, this knowledge gap restricts our understanding of the origins of many infectious diseases and the potential risk of viral emergence in humans. To bridge this gap, systematic surveys across a broad range of wildlife taxa are necessary [28]. In this regard, conducting viral surveillance of specimens intended for the meat trade offers valuable opportunities to screen a wide variety of mammalian species that are often challenging to sample due to their rarity, vulnerability, or protected status [13,14,18,29], and to provide a better understanding of the viruses circulating in wildlife that may come into direct contact with humans via the wild meat supply chain.

Alongside the significant bias in host surveillance, our understanding of the wildlife virome is disproportionately focused on specific viruses. Wildlife pathogen screening typically uses targeted PCR assays, focused on known viruses with reported public health or economic consequences and relies on short genetic sequences instead of complete viral genomes [29]. However, to uncover the true diversity of viruses in wildlife samples, it is imperative to employ unbiased viral detection techniques, regardless of prior knowledge of the host-virus associations. Such unbiased detection methods use recent advancements in next-generation sequencing technologies, providing high-quality genomic sequencing data, even for challenging samples with degraded DNA or RNA, such as processed (e.g., smoked or cooked) wild meat [30–33].

In the present study, we use viral metagenomics to analyse a collection of tissue and swab samples from mammal specimens intended for the wild meat trade in several regions of the DRC and Brussels, Belgium. Our goals are to detect and characterise the viral diversity in these wildlife species and to assess the potential health risks associated with activities related to the wild meat supply chain.

## Materials and methods

### Ethics statement

For compliance with ethical standards for the samples collected at African grocery stores in Brussels, Belgium, we refer to Gombeer et al. (2021) [23]. For permits and ethical clearance of samples collected in the Tshuapa Province, DRC, in 2021, we refer to van Vredendaal et al. (2024) [34]. Samples from the archived collection of the Royal Belgian Institute of Natural Sciences (RBINS) and the University of Antwerp (UAntwerp) were collected between 2010 and 2014, before the Nagoya Protocol was formally implemented in the DRC. However, material transfer agreements (MTA) for these samples were provided by the University of Kisangani’s “Centre de Surveillance de la Biodiversité” (CSB). We did not apply for a CITES export permit as the species identity of the meat was unknown at the time.

### Sample collection and mammal species identification

**Fig 1:**
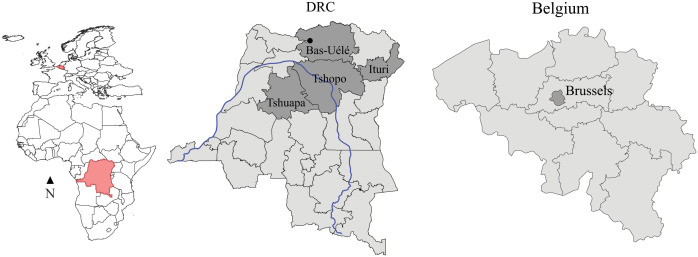
Map showing the provinces in the DRC (middle) and Belgium (right) where samples were collected. On the left, a map of mainland Africa and Europe with the DRC and Belgium marked in red. The provinces where samples were collected are shaded in dark grey in each country. The main course of the Congo River in the DRC in blue. Shapefiles were downloaded from SimpleMaps (https://simplemaps.com/gis/country/cd).

Samples were originally collected from mammals that were intended for the meat trade (i) at African grocery stores in the Matonge neighbourhood in Brussels, Belgium, in 2017 and 2018 (16 samples) [23], (ii) from animals freshly killed by local hunters in Inkanamongo in the Tshuapa Province, DRC, in 2021 (53 samples) [34], and (iii) from archived wild meat samples from carcasses of hunted wildlife and wild meat on display at several rural and urban markets in the provinces of Tshopo, Ituri, and Bas-Uélé (DRC) between 2010 and 2015 (96 samples) (**Fig 1** and **S1 Table**).

Carcasses from the African grocery stores in Brussels were obtained as described in Gombeer et al. (2021) [23] in November − December 2017 and May 2018, and frozen at -20°C. The carcasses were partially unfrozen to collect three pieces of muscle and/or bone marrow, which were stored at -20°C until further processing. The samples collected in Inkanamongo consisted of an oral, a nasal, a rectal, and a urogenital swab for each carcass, which were kept in DNA/RNA Shield in the field for three to five weeks and later transferred to -80°C for long-term storage. The samples collected in the provinces of Tshopo, Ituri, and Bas-Uélé consisted of samples of various tissues from both fresh and processed (smoked or grilled) wild meat, which have been stored in ethanol at room temperature or in RNAlater and frozen at -80°C (**S1 Table**).

DNA was extracted from the samples using the QIAamp DNA Micro Kit or the DNeasy Blood & Tissue Kit (spin column or plate extraction kits, Qiagen), following the manufacturer’s instructions. The mammal species were identified through a multiplex DNA barcoding method, following the protocol outlined by Gaubert et al. (2015) [35], which targets four mitochondrial genes: the cytochrome b gene (*cytb*), the cytochrome *c* oxidase subunit I gene (*COI*), and the ribosomal subunits 12S and 16S. The multiplex PCR products were sequenced using the Oxford Nanopore Technology on a Flongle flow cell. If a target sequence was not recovered, it was re-amplified in a singleplex PCR and Sanger sequenced by Macrogen Europe.

### RNA extraction

RNA from muscle and bone marrow samples collected in Brussels was extracted separately using the Nucleospin RNA Kit (Macherey-Nagel) following the manufacturer’s instructions, eluting with 60 μL RNase-free water. Distinct extracts per specimen were pooled and concentrated to 35 µL using the RNeasy MinElute Cleanup Kit (Qiagen).

Prior to RNA extraction of the samples collected in the Tshuapa Province, we pooled 70 µL of the DNA/RNA Shield medium of each of the four swabs after vortexing. RNA extraction was performed with the QIAmp Viral RNA Mini Kit (Qiagen) following the manufacturer’s instructions but substituting Qiagen’s columns for Zymo-Spin columns IIC (Zymo Research).

The RNA from samples collected from the archival collection was extracted using the Nucleospin RNA Kit (Macherey-Nagel) following the manufacturer’s instructions but without adding β-mercaptoethanol during the lysis step.

### PCR-based virus detection

As an initial viral screening, we conducted multiple PCRs targeting viruses frequently associated with human epidemics, i.e., *Coronaviridae*, *Filoviridae* (e.g., Ebola virus, Marburg viruses), *Flaviviridae* (e.g., hepatitis C virus), *Paramyxoviridae* (e.g., canine distemper virus, measles virus, parainfluenza virus), simian immunodeficiency virus (SIV), and *Orthopoxvirus* (e.g., monkeypox virus) [36]. To screen for RNA viruses, RNA extracts were reverse-transcribed using Maxima Reverse Transcriptase (Thermofisher) following the manufacturer’s instructions and PCRs were carried out on the resulting cDNA. To screen for *Orthopoxvirus*, the DNA extracts were subjected to a quantitative real-time PCR (see **S2 Table** for information on primers and cycling conditions). PCR products were run on a 1.5% agarose gel and, if a band at the expected height was visible, Sanger sequenced at the University of Antwerp VIB sequencing facility.

### Library preparation and next-generation sequencing (NGS)

For library construction, RNA extracts were pooled by mammalian taxa and country of sampling, with each pool containing samples from one to fourteen individuals (**S1 Table**). For each pool, 14–50 µL of RNA extract from each sample was combined to 100–200 µL, which was further concentrated to approximately 40–45 µL using the RNeasy MinElute Cleanup Kit (Qiagen). RNA was then quantified with the Qubit RNA HS Assay Kit on a Qubit 2.0 Fluorometer (Life Technologies). For 22 pools (containing a total of 64 individual samples), no RNA was detectable, and these were omitted for further NGS.

RNA pools were sent to Genewiz (Azenta Life Sciences) for cDNA generation, library preparation, and sequencing using Strand-Specific RNA-Seq. The library preparation included an rRNA depletion step to remove host rRNA from the libraries and thereby relatively increase the yield of viral RNA reads. Libraries were 150 bp paired-end sequenced on an Illumina NovaSeq platform.

### Virus detection and genome assembly

The overall quality of the reads in each library was checked with FastQC v0.11.7 [37]. Subsequently, reads were paired with Trimmomatic v0.36 [38] and (part of) the reads with a low-quality score were discarded, i.e., when the Phred-33 quality score within a window of four bases was below 15 and when the read was less than 50 bp long. To filter out reads originating from the host genome, trimmed reads were aligned against the corresponding host genome (or a phylogenetically closely related host genome in case the respective host genome was not available in the database of the National Center for Biotechnology Information (NCBI) (https://www.ncbi.nlm.nih.gov/)) using the ‘bwa index’ and ‘bwa mem’ algorithm of BWA v0.7.17 [39] (**S3 Table**). The unmapped reads were *de novo* assembled into contigs using the rnaviralSPAdes assembler of SPAdes v3.15.5. Next, the resulting contigs were mapped against the nucleotide (nt) database of NCBI (available as of December 11, 2024) using BLASTn (BLAST+ v2.13.0) with a word size of 16 and an E-value cut-off of 10^-10^. Contig hits with an alignment length of at least 100 bp and a percentage identity higher than 85% were retained. For the remaining contigs, we conducted a protein-level search by mapping the contigs against the NCBI ClusteredNR database (available as of May 28, 2025) using BLASTx with a word size of 3 and an E-value cut-off of 10^-10^. Contig hits with an alignment length of at least 50 amino acids and a percentage identity of 30% were retained. Contigs that matched viral families known to infect terrestrial vertebrates (as classified by the International Committee on Taxonomy of Viruses (ICTV); https://ictv.global/virus-properties) were aligned to the RefSeq whole genome of the species corresponding to the top BLAST hit. The top hit was defined as the match with the highest percentage identity and lowest E-value. If contigs did not map to the RefSeq genome, the original BLAST hit was used as the reference. When the BLAST hit was a protein, the corresponding nucleotide sequence was used instead. Alignments were performed using Geneious Prime v2024.0.3, with the highest sensitivity settings and up to ten iterative refinements. Quality-trimmed paired reads were mapped back to the consensus of the assembled contigs (majority consensus with the reference genome sequence value when there was no coverage) using Bowtie2 v2.4.4 with the local alignment mode ‘--very-sensitive-local’ to assess coverage (i.e., percentage of the reference sequence covered by reads) and depth (i.e., the average number of reads that detected a certain base in the genome), and the resulting consensus (majority consensus with N called when there is no coverage) was blasted against the nt database using BLASTn. The abundance of each virus species in the library was expressed as the number of mapped reads per million total (quality trimmed) reads (RPM). Following Cui et al. (2019, 2023) [40,41], we considered a library to be false positive when the RPM was below 1. In addition, we visually inspected genome coverage to confirm that the reads were covering several regions without overlap. ‘False positives’ were not included in further phylogenetic and recombinant analyses. We did, however, check whether the presence of these viral reads could have been caused by contamination from other viral positive libraries by mapping the viral reads against the viral consensus sequences of the ‘true positive’ pool for the respective viral family (if present) in Geneious Prime. Pie charts for summarising host filtering, taxonomic classification, and viral detection were generated in Rstudio v2024.04.2+764 using the package ‘ggplot2’ v3.5.2 [42].

### Recombination and phylogenetic analyses

The final consensus sequences were assigned to a viral (sub)family based on the lineage information of the top hit in the BLASTn results (**S3 Table**). To verify this preliminary identification of the virus family and further assign the virus to genus and species, we performed recombination and phylogenetic analyses based on the RNA-dependent RNA polymerase (RdRp) gene sequence for RNA viruses (and the *pol* gene encoding reverse transcriptase for retroviruses) and the DNA polymerase gene sequence for DNA viruses, as these replication genes are the most conserved phylogenetic markers for viruses [33,43].

First, these genes were annotated in Geneious Prime using the annotated genomes of one virus exemplar per species in the respective (sub)family (selected based on the Virus Metadata Resource (VMR) from the ICTV) as a reference database and using a 50% identity threshold to transfer reference annotations. The annotated genes were further verified by identifying open reading frames (ORFs) using the ‘Find ORFs’ tool in Geneious Prime.

Next, for each detected virus, we created two alignments: one that included a single sequence from each species in the respective family (the same database as for gene annotation), and another encompassing all viral sequences of the detected genus available in NCBI Virus to cover a broader phylogenetic diversity. However, for the genus *Orthohepadnavirus,* we included only specimens of species that clustered within the same clade as the detected virus in the family-level phylogeny (see Results section) to keep the dataset manageable.

At the family level, the alignment of the replication genes was performed using Translation Align in Geneious Prime at default settings. At the genus level, MAFFT v7.490 was used at default settings in Geneious Prime because not all replication gene fragments in the database were in the same translation frame. Also, short fragments, i.e., fragments shorter than 90% of the total expected length of the replication gene, were removed from the alignment. Subsequently, all positions with gaps were removed using TrimAl v1.2 [44]. For retroviruses of the subfamily *Spumaretrovirinae* (family *Retroviridae*) (see Results section), we also performed recombination and phylogenetic analyses with a shorter *pol* gene sequence (271 bp after trimming and gap removal) to include a substantially larger amount of GenBank sequences into the analyses, i.e., 543 sequences instead of the 34 that were included with the longer *pol* gene fragment.

To test for the presence of recombination, we performed a PHI-test [45] implemented in SplitsTree4 v4.19.2 [46] in combination with the recombination detection methods BootScan (window size 200) [47], Chimaera (variable window size) [48], MaxChi (variable window size) [48], RDP (window size 100 bp) [49], 3Seq [50], and SisScan (window size 200) [51] implemented in RDP4 v4.101 [52]. In case the PHI-test was significant, and a recombination event was detected by at least three detection methods in RDP4, the recombinant region was excluded from the alignment prior to conducting the phylogenetic analyses.

To infer maximum likelihood (ML) trees, we used IQ-TREE v2.2.2.6 [53] with model selection based on the Bayesian Information Criterion (BIC) using ModelFinder [54] and 1,000 bootstrap replicates (**S4 Table**). Pairwise nucleotide *p*-distances (*p* is the proportion of nucleotide positions where the two compared sequences differ) were estimated using MEGA11 v11.0.13 [55] using the gamma parameter and rates among sites as determined by IQ-TREE. Trees were visualised and midpoint-rooted in Rstudio using the packages ‘ape’ v5.5 [56] and ‘ggtree’ v3.0.2 [57]. A bubble plot summarising the viral genera identified in the various read pools was also generated in Rstudio using the packages ‘ggplot2’ v3.5.2.

## Results

### Host identification

The complete collection of mammal samples comprised 165 specimens, which molecular barcoding showed to belong to 41 distinct species of the orders Artiodactyla (11), Carnivora (3), Hyracoidea (1), Macroscelidea (2), Pholidota (1), Primates (13), and Rodentia (10). Only genus-level, not species-level, identification could be determined for 21 samples. Due to inconsistencies in the sample, the host of one specimen (UAC-168) could not be determined (**S1 Table**). The European Nucleotide Archive (ENA) accession numbers corresponding to all sequences generated for host identification are provided in **S1 Table**.

### PCR-based detection of targeted virus families on individual samples

None of the 165 specimens tested positive in the PCR assays targeting viruses of *Coronaviridae*, *Filoviridae*, *Flaviviridae*, *Paramyxoviridae*, or *Orthopoxvirus*. However, all assays are known to miss some members of the targeted virus families or genus. Furthermore, the RNA quantity and quality was below the detection level for at least 65 specimens (as 22 pools of 64 extracts had undetectable RNA levels (see further)). One specimen of Allen’s swamp monkey, *Allenopithecus nigroviridis*, hunted in Tshuapa Province, DRC, tested positive for SIV (family *Retroviridae*, subfamily *Orthoretrovirinae*, genus *Lentivirus*, species *Lentivirus simimdef*). This specimen is included in pool 51 in the metagenomic screening (see further).

### Metagenomic sequencing of sample pools

After species identification and PCR-based screening, specimen samples were pooled into a total of 52 RNA libraries (**S1 Table**). However, for 22 of the 52 pools of RNA extracts, RNA levels were below the detection limit and were omitted from metagenomic analyses. RNA detectability and concentration appeared mostly determined by the storage conditions of the sample and secondarily the level of processing of the meat. Of the 21 pools that consisted solely of extracts of samples of fresh or smoked meat specimens stored in ethanol at room temperature, 18 did not yield detectable RNA, while ‘only’ four out of the ten pools that consisted of samples from smoked/grilled carcasses, but that had been frozen at -20°C, did not contain detectable RNA. All pools containing RNA extracts of at least one frozen sample stored in RNAlater or DNA/RNA Shield, contained detectable RNA (between 5.2 and 114 ng/µL).

**Fig 2:**
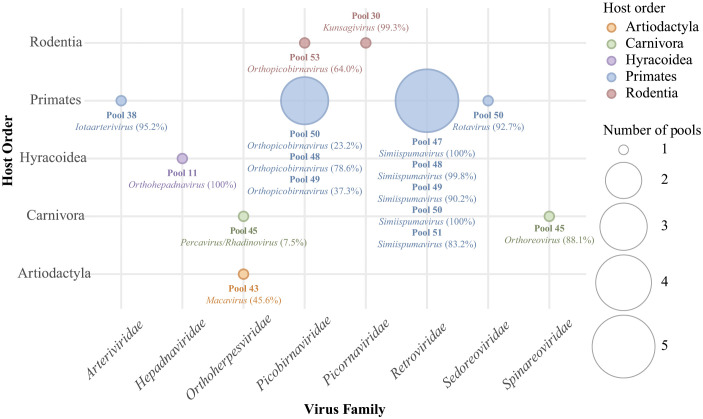
Overview of host-virus associations in the read pools. Each bubble represents a host–virus pair, with bubble size proportional to the number of read pools in which the virus family was detected. Information about the read pool and virus genome coverage (in brackets) is indicated under each corresponding bubble.

For the 30 deep-sequenced RNA pools, covering a total of 101 specimens of 29 mammalian species, the total number of reads produced per sequencing library ranged from 113 to 840 million reads with an average of 195 million reads. After trimming and host reads filtering, the number of reads remaining for *de novo* assembly and subsequent blasting of the contigs to the nt database ranged from 0.2 to 152 million reads with an average of 46 million reads (**S1a** and **S1b Fig**). Most contigs were of eukaryotic or bacterial origin (**S1a** and **S1c Fig**). Among the viral contigs (17,993 contigs), the majority corresponded to bacteriophages (2,924 contigs), unclassified phages, archaeal viruses, and mycoviruses (10,575 contigs), or viruses known to infect only invertebrates (808 contigs) (**S1a**, **S1d****–****S1f Fig**). Only a small fraction of the viral contigs (677 contigs of which 56 were classified as endogenous viral elements) could be assigned to viral families known to infect vertebrate hosts. These were detected in 24 of the 30 libraries, with mapped read counts ranging from 2 to over 3.7 million reads and RPMs spanning from 0.011 to 16,778.355 (**S3 Table** and **S1a Fig**). To reduce the probability of further analysing contaminating viral reads, we focused only on reads of terrestrial vertebrate-associated viruses with an RPM > 1. Using this criterion, we detected viruses in eleven of the 30 pools (**Figs 2**, **S1a**, **S1g**, **S1h**). From the six investigated RNA pools of muscle and bone marrow from smoked carcasses collected in restaurants and shops in Brussels, a vertebrate virus with RPM > 1 was detected in a pool of two De Brazza’s monkey samples. From the nine investigated pools of archived, frozen organ samples from fresh or smoked wild meat carcasses from various locations in the DRC, we detected vertebrate viruses in two pools: one consisting of a tree hyrax sample and one consisting of five rope squirrel samples. No viruses were detected in three pools of tissue samples that had been stored in ethanol at room temperature. In the twelve investigated pools consisting of oral, rectal, urogenital, and nasal swabs of fresh wild meat carcasses, sampled in 2021 in Inkanamongo, DRC (two pools of artiodactyls, two of carnivores, one of macroscelid, two of rodents, five of primates), we detected vertebrate viruses in nine pools (one artiodactyl, one carnivore, one rodent, and all five primate pools). The identified viruses belong to two DNA virus families and six RNA virus families (see details per virus family below). The raw sequence data, i.e., the raw reads produced per sequencing library, have been deposited in the NCBI Sequence Read Archive (SRA) under BioProject accession number PRJNA1247736. The assembled contigs have been deposited in the GenBank database. The SRR and GenBank accession numbers are provided in **S3 Table**. The consensus sequences of the viral genomes or partial genomes derived from the SRA data have been deposited in the ENA database. Their accession numbers are provided in **S6 Table**.

For the detected viruses for which RPM < 1, we only found potential contamination in pool 14 and pool 38. Viral reads belonging to the family *Hepadnaviridae* from these pools mapped against the consensus sequence of the hepadnavirid strain from pool 11 with 99.6% (eighteen in pool 14) and 100% identity (two reads in pool 38) (**S3 Table**). For the other pools with RPM < 1, viral reads either did not have significant matches to reads from other samples or had multiple nucleotide differences with the consensus sequences of ‘true’ positive pools, indicating they were not the result of cross-contamination (**S3 Table**). Additional (deeper) sequencing and/or targeted PCR screening, and the characterisation of the host genome could help determine whether these samples are truly negative and/or endogenous. In pool 51, we detected six viral reads belonging to SIV which all mapped to the *gag* gene (GenBank accession PV553524; SRR accession SRR33015580). Although the RPM was below 1, one specimen included in this pool (EBO1480) tested positive for SIV using a conventional PCR assay targeting a fragment of the *pol* gene (GenBank accession PV553528), which confirms that this pool is indeed positive for this virus.

### Phylogenetic positioning of the detected viruses

**Fig 3:**
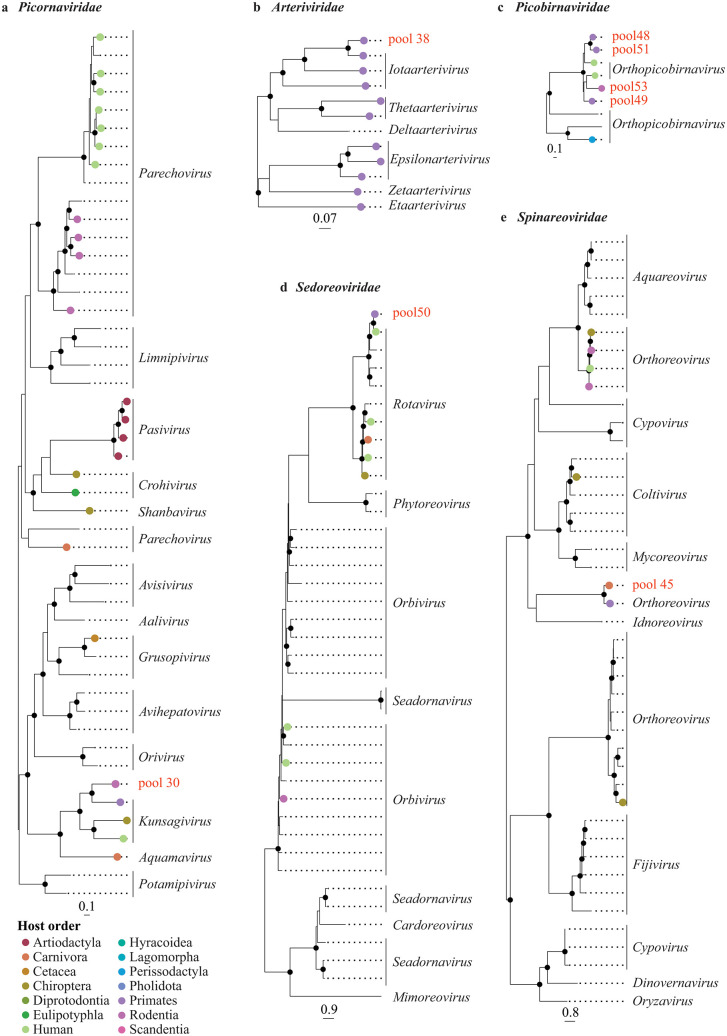
Phylogenetic relationships of members of the kingdom *Orthornavirae*: *Picornaviridae* (a), *Arteriviridae* (b), *Picobirnaviridae* (c), *Sedoreoviridae* (d), and *Spinareoviridae* (e) inferred from the RNA-dependent RNA polymerase gene. Maximum likelihood phylogenetic trees are shown with only well-supported nodes (bootstrap ≥ 85%) indicated by black dots. Scale bar below each tree indicates the mean number of nucleotide substitutions per site. Tip points of strains infecting Mammalia are coloured by host order, with humans distinguished from other primates by a different colour. The tip label for the viral strain detected in the present study is shown in red and labelled by the pool in which it was detected. For the other strains, the tip label represents the respective viral genus.

**Fig 4:**
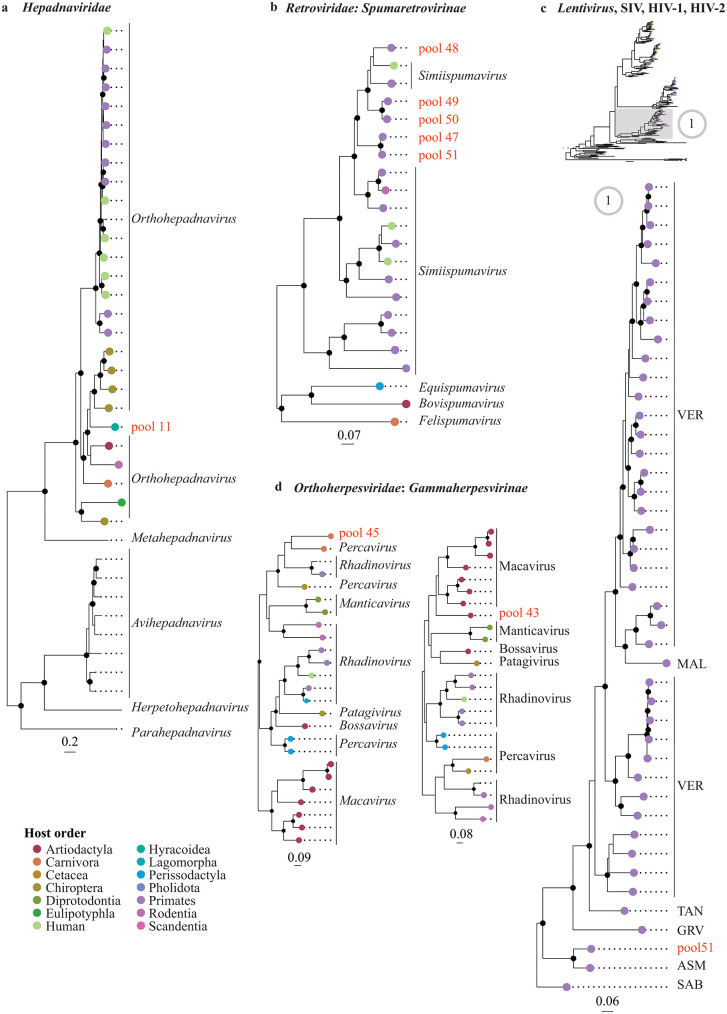
Phylogenetic relationships of members of the kingdom *Pararnavirae* and *Heunggongvirae*: *Hepadnaviridae* (a), *Spumaretrovirinae* (*Retroviridae*) (b), SIV, HIV-1, and HIV-2 (*Lentivirus*, *Orthoretrovirinae*, *Retroviridae*) selected from the curated alignment (Web Alignment) downloaded from https://www.hiv.lanl.gov/ (c), and *Gammaherpesvirinae* (*Orthoherpesviridae*) (d) inferred from the *pol* gene (b, c) and DNA polymerase gene (a, d). Maximum likelihood phylogenetic trees with only well-supported nodes (bootstrap ≥ 85%) indicated by black dots. The scale bar below each tree indicates the mean number of nucleotide substitutions per site. Tip points of strains infecting Mammalia are coloured by host order, with humans distinguished from other primates by a different colour. The tip label for the viral strain detected in the present study is shown in red and labelled by the pool in which it was detected. For the other strains, the tip label represents the respective viral genus (or subtype of SIV in **(c)**).

**Fig 5:**
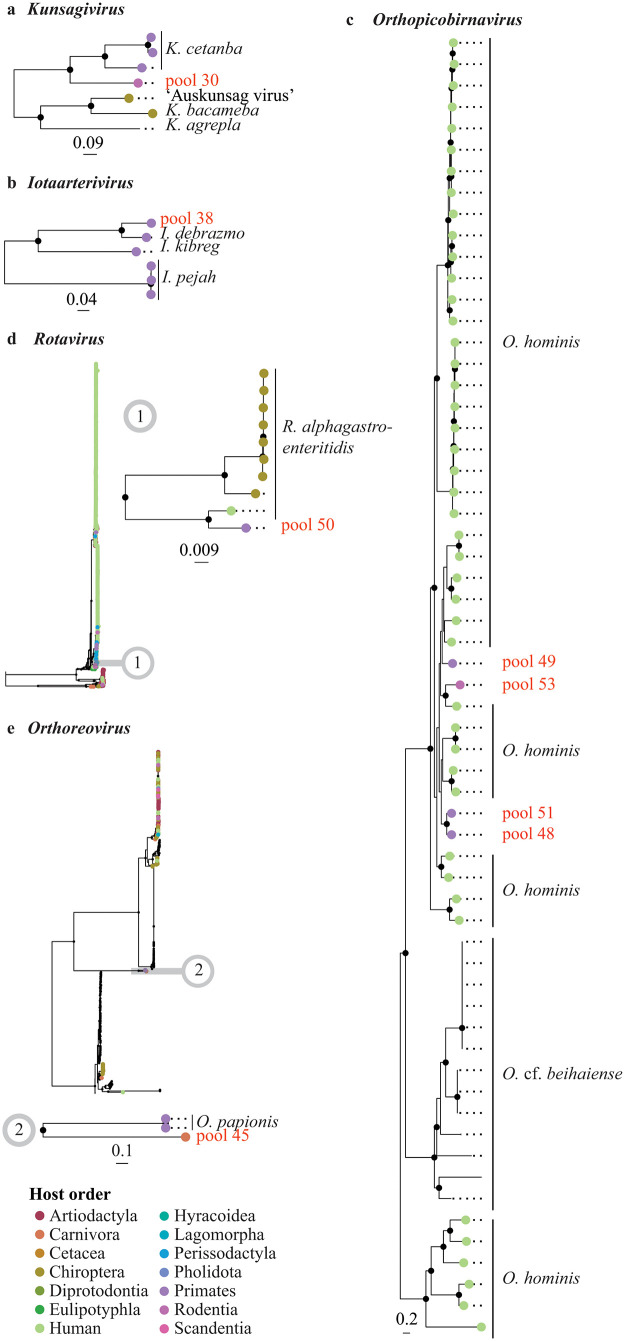
Phylogenetic relationships of members of the kingdom *Orthornavirae*: *Kunsagivirus* (a), *Iotaarterivirus* (b), *Orthopicobirnavirus* (c), *Rotavirus* (d), and *Orthoreovirus* (e) inferred from the RNA-dependent RNA polymerase gene. Maximum likelihood phylogenetic trees are shown with only well-supported nodes (bootstrap ≥ 85%) indicated by black dots. The scale bar below each tree represents the mean number of nucleotide substitutions per site. Tip points of strains infecting Mammalia are coloured by host order, with humans distinguished from other primates by a different colour. The tip label of the viral strain detected in the present study is shown in red and is labelled by the pool it was detected in. For other strains, the tip label represents the respective viral species.

**Fig 6:**
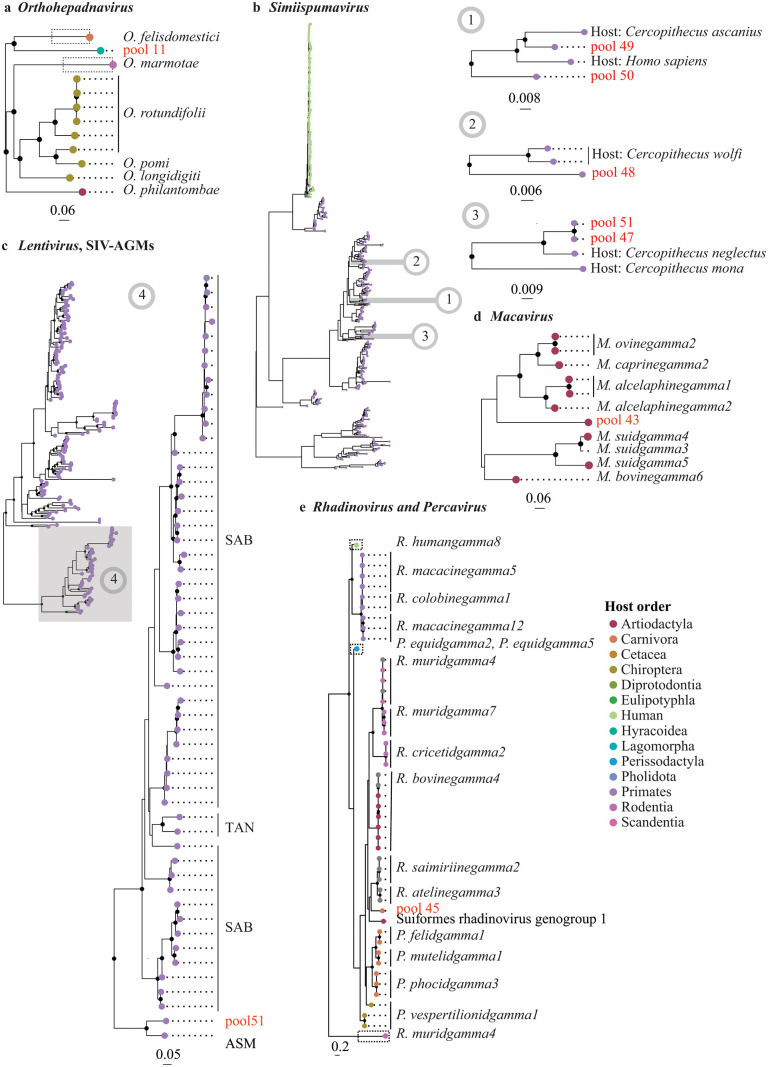
Phylogenetic relationships of members of the kingdom *Pararnavirae* and *Heunggongvirae*: *Orthohepadnavirus* (a), *Simiispumavirus* (b), *Lentivirus* belonging to the clade of SIV-AGMs downloaded from https://www.hiv.lanl.gov/ (c), *Macavirus* (d), and *Rhadinovirus* and *Percavirus* (e) inferred from the *pol* gene (b, c – only a short fragment of 271 bp was used for (b)) and DNA polymerase gene (a, d, e). Maximum likelihood phylogenetic trees are shown with only well-supported nodes (bootstrap ≥ 85%) indicated by black dots. The scale bar below each tree represents the mean number of nucleotide substitutions per site. Tip points of strains infecting Mammalia are coloured by host order, with humans distinguished from other primates by a different colour. The tip label for the viral strain detected in the present study is shown in red and labelled by the pool in which it was detected. For the other strains, the tip label represents the respective viral species (or host species in (b) and subtype of SIV in (c)). Collapsed clades are shown as dotted-line boxes.

We examined the phylogenetic relationships of the detected viruses at two levels: (i) at the family level to determine the virus genus (**Figs 3** and **4**), and (ii) at the genus level to offer a more detailed depiction of the evolutionary intrageneric relationships of the detected viruses (**Figs 5** and **6**).

The recombination analyses (Phi-test, BootScan, Chimaera, MaxChi, RDP, 3Seq, and SisScan) that were performed before each phylogenetic analysis (at both family and genus level) detected significant recombination events, i.e., the significance of the PHI-test and the detection of recombination events by at least three of the other detection methods, in the dataset of arterivirids (pool 38) and orthoreoviruses (pool 45) (**S5 Table**). The recombinant regions were excluded from the alignments prior to conducting the phylogenetic analyses. Note that for the recombination tests at the genus level for the rotavirus in pool 50, we only selected a subset of the sequences (i.e., the sequences within the clade depicted in **Figs 5d** and **S11b**) to maintain a manageable dataset.

### *Arteriviridae* (pool 38)

#### Iotaarterivirus.

An arterivirus belonging to the genus *Iotaarterivirus* (family *Arteriviridae*, subfamily *Simarterivirinae*) was detected (genome coverage 95.2%) in the read pool from the muscle and bone tissue of two De Brazza’s monkeys, which were collected from smoked meat in a shop in Brussels (pool 38) in Gombeer et al. (2021) [23] (**Figs 2**, **3b**, **S2a**, and **S6 Table**). It is closest related to DeBrazza’s monkey arterivirus (species *Iotaarterivirus debrazmo*) (GenBank accession number NC_026509) collected from the same primate species in Cameroon (*p*-distance = 0.147) (**Figs 5b**, **S2b**, and **S7 Table**). A strain of *Iotaarterivirus kibreg* found in another species of *Cercopithecus*, a red-tailed monkey, further clusters with this group.

### *Hepadnaviridae* (pool 11)

#### Orthohepadnavirus.

The strain belonging to the genus *Orthohepadnavirus,* family *Hepadnaviridae*, was detected (full genome coverage) in a frozen, RNAlater-stored liver sample from a fresh tree hyrax carcass from the Tshopo Province (pool 11) (**Figs 2**, **4a**, **S3a**, and **S6 Table**). This is the first time that this viral genus is detected in species of *Dendrohyrax*. In the intrageneric phylogenetic tree (**Figs 6a** and **S3b**), this strain groups with a clade containing members of domesticated cat hepadnavirus (species *Orthohepadnavirus felisdomestici*) infecting domesticated cats from Asia, Europe, North America, South America, and Australia, and with a clade containing members of roundleaf bat hepatitis B virus (species *Orthohepadnavirus rotundifolii*) infecting bats from Africa and Asia, long-fingered bat hepatitis B virus (species *Orthohepadnavirus longidigiti*) and pomona bat hepatitis B virus (species *Orthohepadnavirus pomi*) infecting bats from Asia, and Taï Forest hepatitis B virus (species *Orthohepadnavirus philantombae*) infecting an artiodactyl from Africa. Based on the pairwise nucleotide *p*-distances, the viral strain detected in the present study is closest related to roundleaf bat hepatitis B virus infecting bats of *Hipposideros* and *Rhinolophus* from Gabon (*p*-distance = 0.330–0.333) (**S8a Table**). As a full viral genome (3,188 bp) was recovered from this read pool, we additionally calculated the *p-*distances based on the full genome sequence (**S8b Table**), which shows the smallest *p*-distance between the viral strain detected in the present study and the strain of roundleaf bat hepatitis B virus infecting a bat of *Rhinolophus* from Gabon (*p-*distance = 0.327). According to the species demarcation criterion proposed for hepadnavirids (more than 20% nucleotide sequence divergence of the complete genomes) (*p*-distance > 0.20), the virus identified in this study can be considered a new member of the genus *Orthohepadnavirus,* which we tentatively name ‘Tree hyrax hepadnavirus’.

### *Orthoherpesviridae* (pool 43 and 45)

#### *Percavirus* or *Rhadinovirus.*

A gammaherpesvirin strain (family *Orthoherpesviridae*, subfamily *Gammaherpesvirinae*) was detected in the pooled swab samples of a fresh carcass of an African palm civet (pool 45) from Inkanamongo (genome coverage 7.5%). This is the first time that an orthoherpesvirid is detected in this mammal host (**Figs 2** and **4d**). The detected strain falls phylogenetically within the clade containing members of the genera *Percavirus* and *Rhadinovirus*. Based on the DNA polymerase gene fragment, the lowest *p*-distance is found with members of vespertilionid gammaherpesvirus 1 (species *Percavirus vespertilionidgamma1*) (GenBank accession number KU220026) infecting a bat (*Myotis velifer*) from North America (*p*-distance = 0.347) (**S9 Table**).

#### Macavirus.

Another strain of gammaherpesvirus was detected (genome coverage 45.6%) in the pooled swab samples from fresh carcasses of two blue duikers (pool 43) from Inkanamongo (**Figs 2**, **4d**, and **S6 Table**). This is the first time that an orthoherpesvirid is detected in this host. Based on phylogenetic analyses using fragments of the DNA polymerase (**Figs 6** and **S5**), this strain clusters with members of the genus *Macavirus* (family *Orthoherpesviridae*, subfamily *Gammaherpesvirinae*) infecting artiodactyls from North America and Africa. Based on the pairwise *p*-distances of the DNA polymerase gene fragment, the strain detected here was most similar to a strain of caprine gammaherpesvirus 2 (species *Macavirus caprinegamma2*) (GenBank accession number NC_043059) infecting goats (*p*-distance = 0.290) (**S10 Table**).

### *Picobirnaviridae* (pool 48, 49, 51, and 53)

#### Orthopicobirnavirus.

Four distinct strains belonging to the genus *Orthopicobirnavirus* (family *Picobirnaviridae*) were detected in swab samples of several mammal species collected from fresh carcasses: one in a pool of four Wolf’s or Dent’s monkeys (pool 48) (genome coverage 78.6%), one in a pool of eight red-tailed monkeys (pool 49) (genome coverage 37.3%), one in a pool of two Allen’s swamp monkeys (pool 51) (genome coverage 23.2%), and one in a pool of thirteen northern giant pouched rats and one African brush-tailed porcupine (pool 53) (genome coverage 64.0%) (**Figs 2**, **3c**, **S6a**, and **S6 Table**). All these animals were sampled in Inkanamongo, DRC. According to the phylogenetic analyses using the RdRp gene fragment, all three strains cluster together with human picobirnavirus (species *Orthopicobirnavirus hominis*) from Asia and Europe (**Figs 5c** and **S6b**). All four pools have the smallest *p*-distances to strains of human picobirnavirus from India (see **S11 Table** for *p*-distances).

### *Picornaviridae* (pool 30)

#### Kunsagivirus.

In the pool of frozen, RNAlater preserved liver samples from fresh carcasses of five rope squirrels (*Funisciurus* spp.) from the Tshopo Province (pool 30), we detected a strain belonging to *Kunsagivirus* (genome coverage 99.3%), which is the first time that this viral genus is detected in this mammal genus (**Figs 2**, **3a**, **5a**, and **S6 Table**). According to the phylogenetic analyses, the detected strain clusters with members of kunsagivirus C1 (or bakunsavirus) (species *Kunsagivirus cetanba*) (**Figs 5a**, and **S7**) with the closest sequence similarity to a bakunsavirus infecting a yellow baboon, *Papio cynocephalus*, from Tanzania (GenBank accession number NC_034206; *p*-distance = 0.383) (**S12 Table**). As this is below the cut-off for the species demarcation criterion proposed for the genus *Kunsagivirus* (52% nucleotide difference within the 3 CD gene), we classified the detected strain as belonging to the species *Kunsagivirus cetanba*.

### *Retroviridae* (pool 47, 48, 49, 50, and 51)

#### Simiispumavirus.

Strains of simian foamy virus (SFV) (family *Retroviridae*, subfamily *Spumaretrovirinae*, genus *Simiispumavirus*) were detected in all pools of swab samples from fresh carcasses of primates collected in Inkanamongo: in pool 47 and 51 including Allen’s swamp monkeys (genome coverage 100% and 83.2%, respectively), pool 48 including Wolf’s or Dent’s monkey (genome coverage 99.8%), and pool 49 and 50 including red-tailed monkeys (genome coverage 90.2% and 100%, respectively) (**Figs 2**, **4b**, **6b**, **S8**, and **S6 Table**). This is the first time that SFV is detected in Allen’s swamp monkey and Wolf’s or Dent’s monkey.

Based on the phylogenetic analyses of the short (271 bp) *pol* gene fragment (**Figs 6b** and **S9**), strains of SFV detected in Allen’s swamp monkeys phylogenetically cluster with a strain infecting De Brazza’s monkey and Mona monkey, *Cercopithecus mona*, from the DRC (GenBank accession number MN325128 and MN325151, respectively). As species demarcation criteria for *Retroviridae* include virus-host phylogeny, we tentatively call this strain ‘simian foamy virus Allenopithecus nigroviridis’. Strains of SFV detected in Wolf’s monkey or Dent’s monkey were genetically closely related to SFVs detected in Wolf’s monkey in the DRC (GenBank accession number JX157546 and JX157547), which we here call ‘simian foamy virus Cercopithecus wolfi’. Finally, strains of SFV detected in the red-tailed monkey were genetically closely related to SFVs detected in a red-tailed monkey and human from the DRC (GenBank accession number JX157541 and JX157543, respectively) (**Figs 6b** and **S9**) (see **S13 Table** for *p*-distances). We tentatively call this strain ‘simian foamy virus Cercopithecus ascanius’.

#### Lentivirus.

A strain of SIV was detected in the swab samples from a fresh Allen’s swamp monkey carcass collected in Inkanamongo using a PCR assay with SIV-specific primers, targeting a fragment of the *pol* gene [58]. Although the metagenomic RPM was below 1, we examined the phylogenetic intraspecific relationship of the *pol* gene fragment detected with the PCR assay. Because human lentiviruses, i.e., human immunodeficiency virus 1 (HIV-1) and human immunodeficiency virus 2 (HIV-2), resulted from independent cross-species transmission events of their simian counterpart, SIVs, to humans [59], and to maintain a manageable dataset, we obtained curated sequences of SIV, HIV-1, and HIV-2 at Los Alamos National Laboratory (LANL, https://www.hiv.lanl.gov/), which include subtype information. To assign the detected strain to a clade of SIV, we included sequences from the curated alignment ‘Web Alignments’, additionally including a strain of SIVasm (GenBank accession number KX506893) infecting Allen’s swamp monkey [60]. To further assign the strain to an SIV subtype, we included all sequences from the HIV Sequence Database that belonged to the subtypes within the previously assigned clade. Short fragments, i.e., fragments shorter than 90% of the detected fragment, were removed from the alignment. The detected strain has the closest sequence similarity to a strain of SIVasm (*p*-distance = 0.134) isolated from the same host species from the DRC (GenBank accession number KX506893) (**S14 Table**). Together, these two strains phylogenetically fall within the SIV lineage infecting African green monkeys (AGMs, *Chlorocebus* spp.), which includes SIVsab from green monkeys (*C. sabaeus*), SIVgrv from grivets (*C. aethiops*), SIVtan from tantalus monkeys (*C. tantalus*), SIVmal from malbrouck monkeys (*C. cynosures*), and vervet monkeys (*C. pygerythrus*), and clusters basally to SIVver, SIVmal, SIVtan, and SIVgrv (**Figs 4c**, **6c**, and **S10**).

### *Sedoreoviridae* (pool 50)

#### Rotavirus.

A strain of the genus *Rotavirus* (family *Sedoreoviridae*) (genome coverage 92.7%) was detected in the pooled swab samples from fresh carcasses of two red-tailed monkeys collected in Inkanamongo (pool 50) (**Figs 2**, **3d**, **5d**, **S11a**, and **S6 Table**). This is the first time that this viral genus is detected in red-tailed monkeys. Phylogenetically, this strain belongs to a clade that further includes rotavirus A (species *Rotavirus alphagastroenteritidis*) infecting a human from Kenya (GenBank accession number HM627553), giant-leaf-nosed bats, *Macronycteris gigas*, from Gabon (GenBank accession numbers MN528086, MN551587, MN477236, MN477225, MN528101, MN528075, and MN528116), and a Lander’s horseshoe bat, *Rhinolophus landeri*, from Nigeria (GenBank accession number OK087521) (**Figs 5d** and **S11b**). The smallest *p*-distance was found with the strain infecting a human from Kenya (GenBank accession number HM627553) (*p*-distance = 0.037) (**S15 Table**).

### *Spinareoviridae* (pool 45)

#### Orthoreovirus.

In the read pool of the swab samples from a fresh carcass of an African palm civet from Inkanamongo (pool 45), we detected a strain belonging to the genus *Orthoreovirus* (family *Spinareoviridae*) (genome coverage 88.1%), which is the first time that this viral genus is detected in this host (**Figs 2**, **3e**, and **S6**). The detected strain phylogenetically clusters with baboon orthoreovirus (species *Orthoreovirus papionis*) infecting yellow baboons (GenBank accession numbers NC_015877 and HQ847903: identical sequences not subjected to final NCBI review) with *p*-distance = 0.363 (**Figs 5e**, **S12**, and **S16 Table**). According to the species demarcation criteria proposed for orthoreoviruses (i.e., more than 75% nucleotide sequence identity within a species), the strain detected in the present study represents a new orthoreovirus, which we will here tentatively refer to as ‘Civet orthoreovirus’ (Note that the *p*-distances calculated in the present study are only based on the RdRp gene sequence and that additional analyses based on other gene fragments could result in different nucleotide sequence identity levels).

## Discussion

The wild meat trade supports millions of people in the Afrotropics [13,14,17], but the elevated wildlife extraction, driven by urban and diaspora demand, threatens African wildlife diversity and promotes the (international) spread of animal-borne pathogens.

We conducted a metagenomic viral survey of 101 mammalian samples, grouped into 30 pools and including 29 mammalian species (**S1 Table**). Another 22 pools were omitted from metagenomic analyses due to undetectable RNA levels in the pooled extracts, but molecular barcoding for species identification was successfully performed on the DNA extracts of the individual samples. The total collection of mammal samples of 165 animals belonged to 40 wild African species and one domesticated species. Several of the identified mammal species are listed as endangered, vulnerable, or near threatened by the International Union for Conservation of Nature (IUCN) Red list of Threatened Species and/or listed in Appendix I (i.e., species threatened with extinction) and Appendix II (i.e., not necessarily currently threatened with extinction but may become so unless trade is closely controlled) by the Convention on International Trade in Endangered Species of Wild Fauna and Flora (CITES) (**S17 Table**). The presence of these species in our collection highlights the threat that wildlife hunting and trade pose to African biodiversity. Despite the limited sample size (101 animals, 30 RNA pools) and mammal diversity (29 species of 8 orders) included in the metagenomic surveillance of this study, we detected a high diversity of vertebrate viruses, i.e., sixteen strains from eight families: *Arteriviridae*, *Hepadnaviridae*, *Orthoherpesviridae*, *Picobirnaviridae*, *Picornaviridae*, *Retroviridae*, *Sedoreoviridae*, and *Spinareoviridae*. As we explain below, most of these strains were previously unknown, and phylogenetic relationships indicate many may have zoonotic potential.

In the muscle and bone marrow samples of the smoked meat from two De Brazza’s monkeys sampled at the Matonge neighbourhood in Brussels, we detected a simian arterivirus of the genus *Iotaarterivirus* (family *Arteriviridae*, subfamily *Simarterivirinae*), phylogenetically clustering with DeBrazza’s monkey arterivirus (species *I. debrazmo*). Simarterivirins naturally infect various cercopithecoid primate species (i.e., Old World monkeys), causing subclinical infections. Currently, six genera and eleven recognised monophyletic species are included within this subfamily [61]. Some viruses, such as simian hemorrhagic fever virus (SHFV, genus *Deltaarterivirus*), simian hemorrhagic encephalitis virus (SHEV, genus *Epsilonarterivirus*), and Pebjah virus (PBJV, genus *Iotaarterivirus*) [62], cause lethal simian hemorrhagic fever (SHF) in several species of captive Asian macaques after cross-species transmission from their natural host [62–64]. Though simarterivirins are not known to infect humans, their zoonotic risk is considered high due to (i) the fact that at least three distinct members of simarterivirins have already caused fatal infections in captive macaques; (ii) the close phylogenetic relationship between humans and the cercopithecoid host; and (iii) the human immunological naivety to arterivirids [62–64]. In addition, SHFV’s replication in human cells *in vitro* and high virus titres in infected monkeys suggest that these viruses may not require major adaptations to infect humans [65] and that even minimal exposure could pose risks [61]. The detection of DeBrazza’s monkey arterivirus RNA in Brussels highlights how the international wild meat trade extends the geographic reach of potential zoonotic pathogens and associated public health risks. However, the viral RNA presence alone does not confirm whether the virus was still infectious at the time of transportation.

Orthohepadnaviruses have been found in species of Primates [66–70], Rodentia [71,72], Artiodactyla [73], Carnivora [74], Eulipotyphla [75], and Chiroptera [76–78]. Hepadnavirids in general are considered highly host-specific and to have co-diverged with their host. However, several recombination and cross-species transmissions between human and other primate orthohepadnaviruses suggest a geographic rather than host-specific distribution, highlighting the importance of recent cross-species transmissions in the ecology and evolution of orthohepadnaviruses and their zoonotic potential [66,68,79]. Similarly, orthohepadnaviruses infecting African bats (*Hipposideros* and *Rhinolophus*) and Asian grey shrews cluster by geography rather than host species [75,78], further emphasising local cross-species transmissions in their evolution. The orthohepadnavirus detected in the liver of a fresh tree hyrax from the Tshopo Province is phylogenetically closely related to a strain infecting African bats, indicating that cross-species transmissions may occur across mammalian orders (here Chiroptera and Hyracoidea), as suggested by Nie et al. (2019) [75], and hence possibly also to humans.

This study is the first to report a strain of *Orthohepadnavirus* in a species of *Dendrohyrax*, thereby expanding the known host range of this genus. This study is also only the second study to report on the virome of Hyracoidea, following a report of a simplexvirus (family *Orthoherpesviridae*, subfamily *Alphaherpesvirinae*, genus *Simplexvirus*) infecting captive rock hyraxes of the species *Procavia capensis* (Galeota et al., 2009)). These results highlight the need for ongoing surveillance of understudied wild mammalian species.

Two gammaherpesvirins (family *Orthoherpesviridae*, subfamily *Gammaherpesvirinae*) were detected in swabs from fresh African palm civet and blue duiker carcasses from Inkanamongo. Orthoherpesvirids infect diverse vertebrate hosts, including mammals, birds, and reptiles, usually establishing life-long, latent infections. They are highly host-specific and generally show a long-standing co-evolution with their host, typically causing severe disease only in foetuses, young, or immunocompromised individuals [80,81]. Despite this narrow host range, orthoherpesvirids have been reported to infect distant hosts, causing serious illness and raising concerns about their zoonotic potential [80,82]. The strain detected in the African palm civet phylogenetically clusters with members of *Percavirus* (known to infect perissodactyls and carnivores) and *Rhadinovirus* (known to infect primates, rodents, and artiodactyls). However, the low genome coverage and depth prevent clear genus assignment (**S6 Table**). Given the high host specificity of gammaherpesvirins, it is likely a member of *Percavirus*, the only genus within *Gammaherpesvirinae* known to infect carnivores. The strain detected in the blue duiker phylogenetically clusters with members of *Macavirus*, a genus of gammaherpesvirins infecting artiodactylids.

Viruses of the genus *Orthopicobirnavirus* (family *Picobirnaviridae*) were detected in swabs from fresh carcasses of Wolf’s or Dent’s monkey, red-tailed monkey, Allen’s swamp monkey, and pooled samples of northern giant pouched rat and African brush-tailed porcupine from Inkanamongo. All strains phylogenetically cluster with *Orthopicobirnavirus hominis* from diarrhoeic humans in Asia and Europe (**S6 Fig**) [83,84]. Picobirnavirids are linked to diarrhoea and gastroenteritis in terrestrial mammals and birds, though their pathogenicity is still under debate [85]. Recent research suggests picobirnavirids may be bacteriophages and/or mycophages due to several factors: the majority of viruses has been detected in stool samples; they cannot (yet) be cultured in eukaryotic cell lines or isolated from animal tissues; they are detected in non-animal sources (e.g., wastewater, soil, sewage, permafrost); they are closely related to the protist-infecting family *Partitiviridae*; they show no host-virus phylogenetic congruence; and their genomes contain bacterial genetic motifs [86,87, and references herein].

Although the association of picobirnavirids with vertebrate hosts or rather their microbiota is unclear, significant host switching, including human-animal transmission, has been documented within animal-dominating picobirnavirids [87,88]. The potential spillover of these viruses from animals to humans is also supported by our findings, showing high genetic similarity between picobirnavirids in primates, rodents, and humans. Given the potential link of these viruses to human disease, identifying the true host(s) of picobirnavirids is crucial, which could be done by testing if animal cells, bacteria, or fungi support infection or using immunofluorescence to detect the intracellular presence of viral proteins and RNA in tissue biopsies and enteric microbiomes of potential hosts [86].

A strain of kunsagivirus (family *Picornaviridae*, subfamily *Paavivirinae*, genus *Kunsagivirus*) was detected in the liver from fresh carcasses of rope squirrels belonging to *Funisciurus* from the Tshopo Province, phylogenetically clustering with kunsagivirus C1 (bakunsavirus, species *K. cetanba*) infecting yellow baboons from Tanzania. Three recognised species within the genus *Kunsagivirus* include *K. agrepla* (infecting birds) [89], *K. bacameba* (infecting bats) [90,91], and *K. cetanba* (infecting primates) [92,93]. To the best of our knowledge, our study is the first to report kunsagiviruses in rodents, thereby expanding the host range of this genus. Currently, no diseases are linked to these viruses, warranting further research.

Simian foamy viruses (SFV) (family *Retroviridae*, subfamily *Spumaretrovirinae*, genus *Simiispumavirus*) were detected in all five pools of swabs of cercopithecids from Inkanamongo (Allen’s swamp monkey, red-tailed monkey, Wolf’s monkey or Dent’s monkey). The fact that all sample pools of cercopithecids from Inkanamongo were positive could point to a high SFV prevalence in these non-human primate (NHP) species, consistent with findings of Mouinga-Ondémé and Kazanji (2013) [94] who found a high SFV prevalence in NHPs and primate meat from Gabon.

SFVs naturally cause persistent, latent infection in many NHPs. Phylogenetic analyses show strong correlation between the host and virus phylogenies, supporting a long-term host-virus co-evolution [95–97]. This is also corroborated by the findings in the present study, where the detected SFVs phylogenetically cluster according to the host species (**S9 Fig**).

While NHPs have host-specific SFVs resulting from this codivergence, they can acquire SFVs from other primate hosts, as evidenced by cross-species transmissions and several zoonotic spillovers to humans [95,97,98]. SFVs replicate in oral mucosa, and (persistent) infections have been reported in African people, mostly resulting from hunting-related bites [99–102]. Though zoonotic SFV infections are considered non-pathogenic, recent research in humans from Cameroon links haematological abnormalities and mild to moderate anaemia to infection with simian foamy virus Gorilla gorilla gorilla (species *Simiispumavirus gorgorgor*) [100].

The SFV strains detected in red-tailed monkeys are genetically similar to a strain found in a human from the DRC (GenBank accession number JX157543) [102]. Notably, one strain (pool 49) was genetically more similar to the human-infecting strain than the strains from red-tailed monkeys in the study of Switzer et al. (2012) [102], highlighting the zoonotic potential of SFVs in general and their spillover risk from this simian host. To date, no secondary SFV transmissions between humans have been reported; however, studies are limited to small sample sizes and short study durations [102]. Multiple zoonotic spillovers highlight the need to assess exposure risk, secondary transmissibility, and pathogenicity in humans and their natural hosts [102].

HIV-1 and HIV-2, which originated from cross-species transmission of SIVs from NHPs, demonstrate how such simian retroviruses can become pathogenic and transmissible in humans [59,103]. Spillovers of SIVs to humans likely occurred during primate hunting through contact with infected blood [103,104]. To date, SIVs have been found in at least forty-five NHP species, in general each with a species-specific SIV lineage [104]. However, the genetic diversity of SIVs is complex, encompassing host-virus co-evolution, multiple lineages in some hosts, and recombination between distant lineages [104]. The SIVasm strains found in Allen’s swamp monkeys in this study and the study of Ahuka-Mundeke et al. (2017) [60] cluster together and show close genetic proximity to SIVsab, infecting African Green Monkeys from West Africa. Since the geographic ranges of green monkeys and Allen’s swamp monkeys do not overlap [105], direct cross-species transmission between these primate species cannot be inferred. Further genomic analysis could clarify whether SIVasm represents a new species-specific subtype or if it is a recombination of other SIV lineages [60]. It remains unknown if SIVasm can infect humans.

A rotavirus A (RVA) strain (family *Sedoreoviridae*, genus *Rotavirus*, species *R. alphagastroenteritidis*) was detected in the swabs of red-tailed monkeys from Inkanamongo. Rotaviruses cause diarrhoea-associated morbidity in many bird and mammal species (including humans) and are transmitted by a faecal-oral route. Especially strains of RVA are of great concern for public health, being the global leading cause of diarrhoeal disease and deaths in children younger than five years, with the highest mortality in sub-Saharan Africa [106].

Rotaviruses have a linear dsRNA genome with eleven segments encoding six structural (VP1, VP2, VP3, VP4, VP6, and VP7) and five non-structural (NSP1, NSP2, NSP3, NSP4, and NSP5) proteins. They exhibit high intraspecific genetic diversity due to genome segment reassortment during co-infection of the same host cell by different strains, creating unique genotype constellations. Indeed, there are numerous examples in literature of genotype reassortments, often in combination with transmission between various mammalian species, including humans [107–112].

Based on the phylogeny of the RdRp (VP1) gene, the RVA strain in this study clusters with the rare B10 strain infecting an infant from Kenya [109] and strains found in Lander’s horseshoe bat from Nigeria [110] and giant roundleaf bats from Gabon [112]. Genomic analysis of the B10 strain by Ghosh et al. (2011) [109] suggested a simian origin (simian strain SA11) of eight genome segments (VP2, VP3, VP4, VP7, NSP1, NSP2, NSP3, and NSP5) while the origin of the VP1, VP6, and NSP4 genes could not be traced to any known genotype. Then, in 2021, Simsek et al. (2021) [112] found high nucleotide similarity between the B10 strain and bat RVAs for the VP1, VP6, and NSP4 genes, as well as for VP2-4, NSP1, NSP3, and NSP5 genes, suggesting a bat origin of this human strain. A bat origin was further corroborated by the findings of Kia et al. (2021) [110] who found high nucleotide similarity between the VP1 gene of an RVA strain from a bat from Nigeria and strain B10. However, based on the VP1 gene, the present study shows higher genetic similarity between the strain in red-tailed monkeys and B10 (*p*-distance = 0.037) than between the bat strains from Gabon and Nigeria and B10 (smallest *p-*distance = 0.114), supporting a simian origin as proposed by Ghosh et al. (2011) [109]. Further analyses based on the other segments of the RVA genome (VP2–4, VP6-7, NSP1-5) (**S18 Table**) reveal the closest nucleotide similarity between the B10 strain and the strain in red-tailed monkey based on the VP6, VP7, NSP3, and NSP4 segments. For the other segments, the B10 strain was genetically more similar to strains found in primates (including the strain detected in the present study) than to those found in bats. These results further support a simian rather than bat origin of the B10 strain from the Kenyan infant.

Finally, a strain of the genus *Orthoreovirus* (family *Spinareoviridae*) was detected in the swabs of an African palm civet from Inkanamongo. Orthoreoviruses infect reptiles, birds, and mammals [113]. The strain detected in this study phylogenetically clusters with baboon orthoreovirus (species *O. papionis*), which has been detected in the brains of baboons diagnosed with meningoencephalomyelitis [114,115]. However, according to the species demarcation criteria proposed for orthoreoviruses (i.e., more than 75% nucleotide sequence identity within a species), the strain detected in the present study represents a new species of the genus *Orthoreovirus* (based only on the RdRp gene sequence), here tentatively named ‘Civet orthoreovirus’ (note that strains of orthoreoviruses infecting other carnivores are identified as *O. mammalis* and cluster separate from the ‘Civet orthoreovirus’ strain (**S12 Fig**)). Further research is needed to assess the pathogenic potential of this strain and its ability to infect other mammals besides the African palm civet.

The zoonotic potential of a virus is driven by several factors, such as a high abundance of the wildlife host, a high viral prevalence in the wildlife population, frequent human-wildlife contact, close phylogenetic relationship between the wildlife host and humans, and the ability of a virus to infect a wide taxonomic range of hosts [3]. In this regard, NHPs are of great concern as a source of zoonotic viral transmission due to their close phylogenetic relationship with humans, posing a weak phylogenetic barrier for interspecies viral transmission. In addition, NHPs are highly prevalent in the African wild meat trade [116,117], fostering frequent contact with humans. Indeed, many studies, including this one, report viruses in NHPs, and many viral spillovers from primates to humans have been related to activities in the wild meat supply chain, particularly in sub-Saharan Africa [22,32,116,118]. In the present study, we identified several recognised zoonotic pathogens in NHPs which were genetically closely related to human-infecting strains: a strain of *Orthopicobirnavirus* in Wolf’s monkey or Dent’s monkey, red-tailed monkey, and Allen’s swamp monkey; a strain of *Simiispumavirus* in red-tailed monkey, Allen’s swamp monkey, and Wolf’s or Dent’s monkey; and a strain of *Rotavirus* in red-tailed monkey. Their presence in the carcasses of these wild mammals highlights the potential public health risk from human-wildlife interactions in the wild meat supply chain.

In addition, this study is the first to report on the virome of some mammal species, i.e., tree hyraxes of *Dendrohyrax*, Thomas’s rope squirrel, and Dent’s monkey (although the species identification of the latter is uncertain). We also identified known and novel viruses in certain mammal species for the first time (i.e., an orthohepadnavirus in a tree hyrax of the genus *Dendrohyrax*, a kunsagivirus in rope squirrels of the genus *Funisciurus*, orthoherpesviruses in a blue duiker and African palm civet, an orthoreovirus in an African palm civet, a simiispumavirus in Wolf’s or Dent’s monkey, an orthopicobirnavirus in a Wolf’s or Dent’s monkey, red-tailed monkey, Allen’s swamp monkey, and a pool of African brush-tailed porcupine and pouched rats, and a rotavirus in a red-tailed monkey), thereby expanding their known host range and genetic diversity. Whether or not these mammals present the natural reservoir or an incidental host of these viruses, as well as the pathogenesis and prevalence, remains an open question. Nevertheless, their ability to harbour these viruses, even for a short period of time, suggests that they may play a role in viral transmission and the emergence of new strains.

It is important to note that detecting viral genetic material does not necessarily imply that the virus was still infectious at the time of sampling, especially in cooked and processed meats. However, carcasses likely remain infectious during stages like killing and butchering, as many viruses can survive for some time post-mortem [119–121]. This underscores the zoonotic risks at various levels of the supply chain. Reducing this risk will require implementing culturally appropriate and locally relevant measures, such as ensuring access to water and rubber gloves [7].

This study provides important insights into the diversity of viruses in African wild mammals traded for their meat in the DRC and Belgium using viral metagenomics. However, the detected viral diversity likely underestimates the true diversity of viruses that infect these hunted mammals. First, the viral genomic content can be degraded over time depending on the mode of conservation. For example, samples stored in ethanol at room temperature for over a decade are expected to contain only little intact viral DNA/RNA. Also, genomic degradation by meat processing (e.g., smoking or drying) decreases the chance of viral detection [32]. Indeed, most of our sample pools that consisted of tissue samples stored in ethanol at room temperature for several years (including both tissues from smoked and fresh carcasses) did not yield any detectable RNA, and in the three pools (of the 21) that did yield RNA and were sequenced, no viruses were detected. In contrast, all pools containing RNA extracts of at least one frozen tissue or swab sample stored in RNAlater or DNA/RNA Shield contained detectable RNA (between 5.2 and 114 ng/µL). While the sample size was too small to allow a meaningful comparison of virus detection rates between sample types and storage conditions, it is noteworthy that we detected viruses in eight of the twelve pools from the best stored samples —oral, rectal, urogenital, and nasal swabs collected from fresh carcasses and stored at -80°C soon after sampling—whereas viruses were detected in only one of six pools of smoked carcasses (frozen after sampling), two of nine pools from liver samples stored in RNAlater at 4°C for one year and subsequently at -80°C, and in none of the three sample pools of tissues stored in ethanol at room temperature. However, the fact that an almost complete genome of an arterivirus could be recovered from samples of smoked monkey carcasses imported from Africa to Belgium, indicates that the smoking and grilling process does not necessarily render mammal samples unusable for viral metagenomic screening.

Secondly, virus detection relies on sample type as viral presence is influenced by cellular and tissue tropism, viral properties, and the transmission route. Therefore, future surveillance efforts should, whenever possible, include diverse sample types (i.e., muscle, blood, and various organs and swabs) [122]. Thirdly, our phylogenetic analyses were limited to viral strains in pools with an RPM greater than one, likely underestimating the number of virus-positive pools. This is evidenced by the detection of a few SIV reads in pool 51, which tested positive for SIV using a targeted PCR assay. This highlights the high sensitivity of targeted PCR assays and the need for additional PCR testing on pools with an RPM below one. Moreover, to identify the infected individual(s) within a pool and multiple viral strains, further PCR assays and Sanger sequencing on unpooled samples are needed, using primers that could be designed based on the viral consensus sequences characterised in this study. Finally, our metagenomic analyses did not always yield complete viral genomes. Deeper sequencing or PCR using strain-specific primers could fill gaps in the genome.

## Conclusion

Despite a relatively limited sample size of 101 samples of 29 mammal species divided over 30 RNA pools, this metagenomic viral survey revealed a high detection rate and diversity of viruses present in rarely investigated African wild mammals involved in the wild meat trade. Many of the detected viral strains were previously undescribed and may have zoonotic potential. The study highlights the high risk of exposure to various viruses when handling wild mammals.

## Supporting information

S1 Table Detailed information on the samples collected in Brussels and several provinces in the DRC. (XLSX)

S2 Table Information on PCR-based virus detection. (XLSX)

S3 Table Read processing and the detection of viral reads. (XLSX)

S4 Table Best-fitting substitution models based on the Bayesian Information Criterion (BIC) and gamma shape alpha using ModelFinder in IQ-TREE v2.2.2.6. (XLSX)

S5 Table Results from the Phi-test (SplitsTree4) and recombination detection with methods in RDP4. (XLSX)

S6 Table Genome coverage and depth of detected viral sequences with RPM ≥ 1. (XLSX)

S7 Table Pairwise nucleotide *p*-distance estimated based on the RNA-dependent RNA polymerase gene for *Iotaarterivirus.* For each strain that is included in the analysis, the GenBank accession (or pool) number, virus name, country, and host are given (when available from NCBI Virus). Only *p*-distances from the strain found in the present study are shown and ordered by *p*-distance.
(XLSX)

S8 Table Pairwise nucleotide *p*-distance estimated based on the DNA polymerase gene (a) and full genome for *Orthohepadnavirus* (b). For each strain that is included in the analysis, the GenBank accession (or pool) number, virus name, country, and host are given (when available from NCBI Virus). Only *p*-distances from the strain found in the present study are shown and ordered by *p*-distance.
(XLSX)

S9 Table Pairwise nucleotide *p*-distance estimated based on the DNA polymerase fragment for Percavirus/Rhadinovirus. For each strain that is included in the analysis, the GenBank accession (or pool) number, virus name, country, and host are given (when available from NCBI Virus). Only *p*-distances from the strain found in the present study are shown and ordered by *p*-distance.
(XLSX)

S10 Table Pairwise nucleotide *p*-distance estimated based on the DNA polymerase fragment for *Macavirus.* For each strain that is included in the analysis, the GenBank accession (or pool) number, virus name, country, and host are given (when available from NCBI Virus). Only *p*-distances from the strain found in the present study are shown and ordered by *p*-distance.
(XLSX)

S11 Table Pairwise nucleotide *p*-distance estimated based on the RNA-dependent RNA polymerase fragment for *Orthopicobirnavirus.* For each strain that is included in the analysis, the GenBank accession (or pool) number, virus name, country, and host are given (when available from NCBI Virus). Only *p*-distances from the strains found in the present study are shown and ordered by *p*-distance.
(XLSX)

S12 Table Pairwise nucleotide *p*-distance estimated based on the RNA-dependent RNA polymerase fragment for *Kunsagivirus.* For each strain that is included in the analysis, the GenBank accession (or pool) number, virus name, country, and host are given (when available from NCBI Virus). Only *p*-distances from the strain found in the present study are shown and ordered by *p*-distance.
(XLSX)

S13 Table Pairwise nucleotide *p*-distance estimated for *Simiispumavirus* based on the *pol* gene fragment (a) and a shorter *pol* gene fragment to include more GenBank sequences (b). For each strain that is included in the analysis, the GenBank accession (or pool) number, virus name, country, and host are given (when available from NCBI Virus). Only *p*-distances from the strains found in the present study are shown and ordered by *p*-distance.
(XLSX)

S14 Table Pairwise nucleotide *p*-distance estimated based on the RNA-dependent RNA polymerase fragment for *Lentivirus* (SIVagm). For each strain that is included in the analysis, the subtype (as assigned by the staff at the HIV sequence database (https://www.hiv.lanl.gov/)), GenBank accession (or pool) number, virus name, country, and host are given (when available from NCBI Virus). Only *p*-distances from the strain found in the present study are shown and ordered by *p-*distance.
(XLSX)

S15 Table Pairwise nucleotide *p*-distance estimated based on the RNA-dependent RNA polymerase fragment for *Rotavirus.* For each strain that is included in the analysis, the GenBank accession (or pool) number, virus name, country, and host are given (when available from NCBI Virus). Only *p*-distances from the strain found in the present study are shown and ordered by *p-*distance.
(XLSX)

S16 Table Pairwise nucleotide *p*-distance estimated based on the RNA-dependent RNA polymerase fragment for *Orthoreovirus.* For each strain that is included in the analysis, the GenBank accession (or pool) number, virus name, country, and host are given (when available from NCBI Virus). Only *p*-distances from the strain found in the present study are shown and ordered by *p-*distance.
(XLSX)

S17 Table Overview of the molecularly identified host taxa with information on their status on both the International Union for Conservation of Nature and Natural Resources (IUCN) Red List and the Convention on International Trade in Endangered Species of Wild Fauna and Flora (CITES) appendices. (XLSX)

S18 Table Pairwise nucleotide *p*-distances estimated based on the VP2 (a), VP3 (b), VP4 (c), VP6 (d), VP7 (e), NSP1 (f), NSP2 (g), NSP3 (h), NSP4 (i), NSP5 (j) fragment for *Rotavirus.* For each strain that is included in the analysis, the GenBank accession (or pool) number, virus name, country, and host are given (when available from NCBI Virus). Only *p*-distances from the strain of RVA (strain B10) infecting an infant from Kenya (GenBank accession numbers HM627554 (VP2), HM627555 (VP3), HM627556 (VP4), HM627557 (VP6), HM627559 (VP7), HM627560 (NSP1), HM627561 (NSP2), HM627561 (NSP3), HM627562 (NSP4), and HM627563 (NSP5)) are shown and ordered by *p-*distance. The strain that was detected in *Cercopithecus ascanius* in the present study in red.
(XLSX)

S1 Fig Summary of read filtering, taxonomic classification, and viral detection in each read pool. In a, all data is combined across all sequenced RNA sample pools. In b-h, data is displayed per RNA sample pool separately. Number of quality-trimmed reads (in millions) removed during host filtering, and the number of remaining reads available for downstream viral analyses (upper left chart in a; b); number of contigs with a top BLAST hit to archaea, bacteria, eukaryotes, and viruses in combined BLASTn and BLASTx analyses (contigs mapping to unclassified accession numbers in grey) (lower left chart in a; c); number of viral contigs with BLAST hits according to known viral host range (number of contigs classified as endogenous viral elements (EVE) are indicated in red) with (upper pie chart) and without (lower pie chart) unclassified viruses (upper and lower middle charts in a; d-f); and number of contigs (upper pie chart) and reads (lower pie chart) per terrestrial vertebrate viral family detected in each pool (viral families were excluded if contigs did not map to the viral reference, if the consensus of the mapped reads corresponded to non-viral BLAST hits, or if BLAST hits belonged to subfamilies or genera not known to infect terrestrial vertebrates) (upper and lower right charts in a; g-h).
(EPS)

S2 Fig Phylogenetic relationships of members of *Arteriviridae* (a) and *Iotaarterivirus* (b) inferred from the RNA-dependent RNA polymerase gene. Maximum likelihood phylogenetic trees are shown with only well-supported nodes (bootstrap ≥ 85%) indicated by black dots. The scale bar indicates the mean number of nucleotide substitutions per site. For each strain that is included in the analysis, the GenBank accession (or pool) number, virus name, genus (for (a)), country, and host are given (when available from NCBI Virus). Tip points of strains infecting Mammalia are coloured by host order, with humans distinguished from other primates by a different colour and squares next to each tip label are coloured by continent. The tip label for the viral strain detected in the present study is shown in red and labelled by the pool in which it was detected. Note that the continent represents the location of sampling and does not imply that samples were collected from wild animals. The origin of the samples that were collected in Brussels in the present study were assigned to Africa as the meat was imported from this continent.
(EPS)

S3 Fig Phylogenetic relationships of members of *Hepadnaviridae* (a) and *Orthohepadnavirus* (b) inferred from the DNA polymerase gene. Maximum likelihood phylogenetic trees are shown with only well-supported nodes (bootstrap ≥ 85%) indicated by black dots. The scale bar indicates the mean number of nucleotide substitutions per site. For the phylogenetic tree of *Orthohepadnavirus* (b) we only included specimens of species that phylogenetically clustered in the same clade as the detected virus in the family-level phylogeny (framed in grey in (a)) to maintain a manageable dataset. For each strain that is included in the analysis, the GenBank accession (or pool) number, virus name, genus (for (a)), country, and host are given (when available from NCBI Virus). Tip points of strains infecting Mammalia are coloured by host order, with humans distinguished from other primates by a different colour and squares next to each tip label are coloured by continent. The tip label for the viral strain detected in the present study is shown in red and labelled by the pool in which it was detected. Note that the continent represents the location of sampling and does not imply that samples were collected from wild animals.
(EPS)

S4 Fig Phylogenetic relationships of members of *Gammaherpesvirinae* (*Orthoherpesviridae*) inferred from the DNA polymerase gene. Maximum likelihood phylogenetic trees are shown with only well-supported nodes (bootstrap ≥ 85%) indicated by black dots. The scale bar indicates the mean number of nucleotide substitutions per site. For each strain that is included in the analysis, the GenBank accession (or pool) number, virus name, genus (for (a)), country, and host are given (when available from NCBI Virus). Tip points of strains infecting Mammalia are coloured by host order, with humans distinguished from other primates by a different colour, and while squares next to each tip label are coloured by continent. The tip label for the viral strain detected in the present study is shown in red and labelled by the pool in which it was detected. Note that the continent represents the location of sampling and does not imply that samples were collected from wild animals.
(EPS)

S5 Fig Phylogenetic relationships of members of *Macavirus* (a) and *Percavirus*/*Rhadinovirus* (b) inferred from the DNA polymerase gene. Maximum likelihood phylogenetic trees are shown with only well-supported nodes (bootstrap ≥ 85%) indicated by black dots. The scale bar indicates the mean number of nucleotide substitutions per site. For each strain that is included in the analysis, the GenBank accession (or pool) number, virus name, genus (for (a)), country, and host are given (when available from NCBI Virus). Tip points of strains infecting Mammalia are coloured by host order, with humans distinguished from other primates by a different colour, and while squares next to each tip label are coloured by continent. The tip label for the viral strain detected in the present study is shown in red and labelled by the pool in which it was detected. Collapsed clades are shown as dotted-line boxes.
(EPS)

S6 Fig Phylogenetic relationships of members of *Picobirnaviridae* (a) and *Orthopicobirnavirus* (b) inferred from the RNA-dependent RNA polymerase gene. Maximum likelihood phylogenetic trees are shown with only well-supported nodes (bootstrap ≥ 85%) indicated by black dots. The scale bar indicates the mean number of nucleotide substitutions per site. For each strain that is included in the analysis, the GenBank accession (or pool) number, virus name, genus (for (a)), country, and host are given (when available from NCBI Virus). Tip points of strains infecting Mammalia are coloured by host order, with humans distinguished from other primates by a different colour and squares next to each tip label are coloured by continent. The tip label for the viral strain detected in the present study is shown in red and labelled by the pool in which it was detected. Note that the continent represents the location of sampling and does not imply that samples were collected from wild animals.
(EPS)

S7 Fig Phylogenetic relationships of members of *Picornaviridae* (a) and *Kunsagivirus* (b) inferred from the RNA-dependent RNA polymerase gene. Maximum likelihood phylogenetic trees are shown with only well-supported nodes (bootstrap ≥ 85%) indicated by black dots. The scale bar indicates the mean number of nucleotide substitutions per site. For each strain that is included in the analysis, the GenBank accession (or pool) number, virus name, genus (for (a)), country, and host are given (when available from NCBI Virus). Tip points of strains infecting Mammalia are coloured by host order, with humans distinguished from other primates by a different colour and squares next to each tip label are coloured by continent. The tip label for the viral strain detected in the present study is shown in red and labelled by the pool in which it was detected. Note that the continent represents the location of sampling and does not imply that samples were collected from wild animals.
(EPS)

S8 Fig Phylogenetic relationships of members of *Retroviridae* (a) and *Simiispumavirus* (b) inferred from the *pol* gene. Maximum likelihood phylogenetic trees are shown with only well-supported nodes (bootstrap ≥ 85%) indicated by black dots. The scale bar indicates the mean number of nucleotide substitutions per site. For each strain that is included in the analysis, the GenBank accession (or pool) number, virus name, genus (for (a)), country, and host are given (when available from NCBI Virus). Tip points of strains infecting Mammalia are coloured by host order, with humans distinguished from other primates by a different colour and squares next to each tip label are coloured by continent. The tip label for the viral strain detected in the present study is shown in red and labelled by the pool in which it was detected. Note that the continent represents the location of sampling and does not imply that samples were collected from wild animals.
(EPS)

S9 Fig Phylogenetic relationships of members of *Simiispumavirus* inferred from a short (271 bp) fragment of the *pol* gene. Maximum likelihood phylogenetic trees with only well-supported nodes (bootstrap ≥ 85%) indicated by black dots. The scale bar indicates the mean number of nucleotide substitutions per site. For each strain that is included in the analysis, the GenBank accession (or pool) number, virus name, genus (for (a)), country, and host are given (when available from NCBI Virus). Tip points of strains infecting Mammalia are coloured by host order, with humans distinguished from other primates by a different colour and squares next to each tip label are coloured by continent. The tip label for the viral strain detected in the present study is shown in red and labelled by the pool in which it was detected. Note that the continent represents the location of sampling and does not imply that samples were collected from wild animals.
(EPS)

S10 Fig Phylogenetic relationships of members of SIV, HIV-1, HIV-2 (genus *Lentivirus*) selected from the curated alignment (Web Alignments) downloaded from https://www.hiv.lanl.gov/ (a, b) and of all members from the subtypes included within SIV-AGMs downloaded from https://www.hiv.lanl.gov/ (c, d), both inferred from the *pol* gene. Both phylogenetic trees also include the sequence of SIV infecting *Allenopithecus nigroviridis* from Ahuka-Mundeke et al. (2017) [60] (accession number KX506893). Maximum likelihood phylogenetic trees are shown with only well-supported nodes (bootstrap ≥ 85%) indicated by black dots. The scale bar indicates the mean number of nucleotide substitutions per site. For each strain that is included in the analysis, the subtype (as assigned by the staff at the HIV sequence database (https://www.hiv.lanl.gov/)) is indicated (a, c). In (b) and (d) the GenBank accession (or pool) number, virus name, country, and host are also given (when available from NCBI Virus). Tip points of strains infecting Mammalia are coloured by host order, with humans distinguished from other primates by a different colour and squares next to each tip label are coloured by continent. The tip label for the viral strain detected in the present study is shown in red and labelled by the pool in which it was detected. In (b) a subset of the phylogenetic tree framed in grey in (a) is magnified. In (d) a subset of the phylogenetic tree in grey in (c) is magnified. Note that the continent represents the location of sampling and does not imply that samples were collected from wild animals.
(EPS)

S11 Fig Phylogenetic relationships of members of *Sedoreoviridae* (a) and *Rotavirus* (b) inferred from RNA-dependent RNA polymerase. Maximum likelihood phylogenetic trees are shown with only well-supported nodes (bootstrap ≥ 85%) indicated by black dots. The scale bar indicates the mean number of nucleotide substitutions per site. For each strain that is included in the analysis, the GenBank accession (or pool) number, virus name, genus (for (a)), country, and host are given (when available from NCBI Virus). Tip points of strains infecting Mammalia are coloured by host order, with humans distinguished from other primates by a different colour and squares next to each tip label are coloured by continent. The tip label for the viral strain detected in the present study is shown in red and labelled by the pool in which it was detected. In (b) a subset of the entire phylogenetic tree (marked with a grey arrow at the left) is shown magnified (support values and tip labels are omitted in the entire phylogenetic tree to increase readability). Note that the continent represents the location of sampling and does not imply that samples were collected from wild animals.
(EPS)

S12 Fig Phylogenetic relationships of members of *Spinareoviridae* (a) and *Orthoreovirus* (b) inferred from RNA-dependent RNA polymerase. Maximum likelihood phylogenetic trees are shown with only well-supported nodes (bootstrap ≥ 85%) indicated by black dots. The scale bar indicates the mean number of nucleotide substitutions per site. For each strain that is included in the analysis, the GenBank accession (or pool) number, virus name, genus (for (a)), country, and host are given (when available from NCBI Virus). Tip points of strains infecting Mammalia are coloured by host order, with humans distinguished from other primates by a different colour and squares next to each tip label are coloured by continent. The tip label for the viral strain detected in the present study is shown in red and labelled by the pool in which it was detected. In (b) a subset of the entire phylogenetic tree (marked with a grey arrow at the right) is shown magnified (support values and tip labels are omitted in the entire phylogenetic tree to increase readability). Note that the continent represents the location of sampling and does not imply that samples were collected from wild animals.
(EPS)
